# Not only acute respiratory failure: COVID-19 and the post-intubation/tracheostomy upper airways lesions^†^

**DOI:** 10.3389/fsurg.2023.1150254

**Published:** 2023-03-31

**Authors:** Debora Brascia, Angela De Palma, Mirko Girolamo Cantatore, Ondina Pizzuto, Francesca Signore, Doroty Sampietro, Mariangela Valentini, Marcella Genualdo, Giuseppe Marulli

**Affiliations:** Unit of Thoracic Surgery, Department of Precision and Regenerative Medicine and Ionian Area (DiMePre-J), University of Bari “Aldo Moro”, Bari, Italy

**Keywords:** COVID-19, tracheal stenosis, tracheoesophageal fistula, tracheal surgery, endoscopy

## Abstract

**Background:**

An increasing number of patients have been subjected to prolonged invasive mechanical ventilation due to COVID-19 infection, leading to a significant number of post-intubation/tracheostomy (PI/T) upper airways lesions. The purpose of this study is to report our early experience in endoscopic and/or surgical management of PI/T upper airways injuries of patients surviving COVID-19 critical illness.

**Materials and Methods:**

We prospectively collected data from patients referred to our Thoracic Surgery Unit from March 2020 to February 2022. All patients with suspected or documented PI/T tracheal injuries were evaluated with neck and chest computed tomography and bronchoscopy.

**Results:**

Thirteen patients (8 males, 5 females) were included; of these, 10 (76.9%) patients presented with tracheal/laryngotracheal stenosis, 2 (15.4%) with tracheoesophageal fistula (TEF) and 1 (7.7%) with concomitant TEF and stenosis. Age ranged from 37 to 76 years. Three patients with TEF underwent surgical repair by double layer suture of oesophageal defect associated with tracheal resection/anastomosis (1 case) or direct membranous tracheal wall suture (2 cases) and protective tracheostomy with T-tube insertion. One patient underwent redo-surgery after primary failure of oesophageal repair. Among 10 patients with stenosis, two (20.0%) underwent primary laryngotracheal resection/anastomosis, two (20.0%) had undergone multiple endoscopic interventions before referral to our Centre and, at arrival, one underwent emergency tracheostomy and T-tube positioning and one a removal of a previously positioned endotracheal nitinol stent for stenosis/granulation followed by initial laser dilatation and, finally, tracheal resection/anastomosis. Six (60.0%) patients were initially treated with rigid bronchoscopy procedures (laser and/or dilatation). Post-treatment relapse was experienced in 5 (50.0%) cases, requiring repeated rigid bronchoscopy procedures in 1 (10.0%) for definitive resolution of the stenosis and surgery (tracheal resection/anastomosis) in 4 (40.0%).

**Conclusions:**

Endoscopic and surgical treatment is curative in the majority of patients and should always be considered in PI/T upper airways lesions after COVID-19 illness.

## Introduction

The novel coronavirus disease 2019 (COVID-19) is a pandemic caused by the novel severe acute respiratory syndrome coronavirus 2 (SARS-CoV-2). First cases were first reported in Wuhan, China in December 2019 and, since that time, the coronavirus outbreak was declared by the World Health Organization (WHO) as a public health emergency of international concern.

The clinical presentation of COVID-19 is highly heterogeneous, ranging from asymptomatic to severe respiratory failure, requiring invasive mechanical ventilation (IMV) for an average of 9.8%–15.2% of patients (1). Along with this increased need for IMV, an occult, silent and parallel pandemic has raised for surgeons. The mechanic and ischemic damage caused by intubation and tracheostomy on the tracheal wall is a well-known risk factor for the production of fibrotic tracheal scarring (2–4). Moreover, the SARS-CoV-2 has proved to worsen this ischemic tracheal and oesophageal mucosa damage by causing a prothrombotic and antifibrinolytic state, by producing microvascular injury and necrosis and by requiring chronic high dose systemic steroids use (5–7). Prolonged need for IMV in COVID-19 patients, along with the intrinsic capacity of the virus itself to damage the tracheal and oesophageal mucosa, have led to an increased incidence in post-intubation/tracheostomy (PI/T) upper airways lesions, including tracheal stenosis, tracheomalacia and tracheoesophageal fistulas. For this reason, the tracheal surgeon has to become familiar with tracheal lesions resulting from intubation/tracheostomy and with their treatment.

So far, most of the published literature on the COVID-19 PI/T upper airways lesions is anecdotal and mostly made up of case reports (8). We present our case series of COVID-19 patients who developed PI/T lesions after prolonged IMV and were treated at our Institution during the pandemic period.

## Materials and methods

This is a prospective analysis on patients with documented PI/T tracheal injuries after intubation and invasive mechanical ventilation (IMV) due to respiratory failure resulting from SARS-CoV-2 infection who underwent endoscopic or open tracheal/oesophageal surgery between March 2020 and February 2022 in our Thoracic Surgery Unit. All patients were diagnosed with COVID-19 using real-time reverse transcription polymerase chain reaction (RT-PCR) on nasopharyngeal swab.

All patients with suspected or documented PI/T lesions were evaluated with CT of the neck and chest, flexible fiberoptic bronchoscopy and oesophagogastroscopy in case of suspected TEF. The features data were prospectively collected, including patients' demographics (age, body mass index, and comorbidities), length of ICU stay, duration of the endotracheal intubation, time from endotracheal intubation to tracheostomy, duration of tracheostomy and the characteristics of the PI/T lesions (type, size, and localization of the stenosis, categories of the endoscopic interventions and surgeries applied).

Before each operation, nasopharyngeal swabs were taken from the patients for SARS-CoV-2 RT-PCR testing, and only when patients tested negative, they underwent surgery. In only one patient surgery was performed even with a positive SARS-CoV-2 RT-PCR swab; this was the case of a seropositive myasthenia gravis (MGFA class IIB) patient with an ARDS due to COVID-19 infection, coupled with respiratory muscle failure, diagnosed with a TEF of 3.5 cm during her ICU stay. In this case, we considered the emergent surgical repair because of the critical clinical condition of the patient in which prolonged mechanical ventilation was expected. After initial flexible fiberoptic bronchoscopy, type, localization and residual tracheal lumen of the stenosis were assessed and the adequate treatment planned. Endoscopic treatment was considered as the first therapeutic option for those patients with simple web-like stenosis, with a cranio-caudal extent < 1 cm and without any damage to the cartilages, as a bridge-to-surgery option to manage symptomatic emergencies or for patients not eligible for surgery due to poor overall health status and comorbidities or with serious tracheomalacia. Surgery was chosen as first therapeutic option in high grade complex stenosis, with cranio-caudal extension >1 cm, intramural involvement and/or laryngo-tracheal framework impairment or in case of failure of multiple endoscopic procedures.

All patients with TEF, after endoscopic diagnosis was confirmed, underwent urgent surgical repair. Only in one case, surgical repair was delayed because of the priority for the patient, who was 20 weeks pregnant, to undergo prior abortion because of a malformed foetus.

Additionally, specimens of the trachea resected from the patients who underwent tracheal surgery were sent for pathologic examination and were compared with other similar tracheal specimens resected from patients with tracheal stenosis non-COVID-related to assess possible similarities or differences.

### Endoscopic procedures

The rigid bronchoscope was inserted under general anaesthesia and the patient was ventilated through its ventilating channel. The stenosis was radially incised in two or three points with thulium and diode laser thus, the stenotic area was dilated with coring manoeuvres using rigid bronchoscopes with increasing diameters or Savary catheters. At the end of the procedure, an orotracheal tube was placed for 20 min for haemostasis and to stabilize dilatation.

### Surgical procedure: tracheal resection and anastomosis

The surgical principles of tracheal resection and anastomosis (TR/A) have been already described (8). In our experience, after the two 2/0 polyglactin (Vicryl; Ethicon, Inc., Somerville, NJ, USA) traction sutures were placed in the lower tracheal tract and to the larynx, respectively, the posterior anastomosis was carried out first through a 4/0 polydioxanone (PDS; Ethicon, Inc., Somerville, NJ, USA) continuous suture on the membranous pars; soon after, the latero-anterior cartilaginous pars was sutured with interrupted 3/0 Vicryl stitches. The traction sutures were then tied together to release tensions on the anastomosis and pretracheal muscle flaps are used to cover the anastomosis.

### Surgical procedure: closure of the tracheal and oesophageal defects for tracheoesophageal fistula

The surgical principles of primary closure of the tracheal and oesophageal defects for acquired tracheoesophageal fistula have been already described (9). A gastrostomy and a jejunostomy tube should be positioned to allow both enteral feeding and gastric content drainage. Surgery should be delayed until weaning from mechanical ventilation. Postintubation fistulae are usually located in the cervical trachea, thus cervicotomy is the most frequent surgical approach. Trachea and oesophagus were dissected at the location of the fistula, and then closure of the membranous tracheal defect was accomplished directly using interrupted sutures of 4/0 PDS. The oesophageal defect was closed in two layers: the inner oesophageal mucosal layer was closed with a running suture, inverting the edges of the defect into the lumen, followed by closure of the outer oesophageal muscle over the mucosal layer with interrupted 4/0 polydioxanone sutures. A pedicled flap of pretracheal muscles or of sternocleidomastoid muscle was interposed between the oesophagus and the trachea.

### Follow-up

In patients undergoing tracheal resection and anastomosis, control flexible bronchoscopies were done under conscious sedation at postoperative day 1 and day 7 to control the anastomotic suture line. In the TEFs cases, before resuming oral diet, all patients underwent contrast radiography to confirm healing of the oesophagus.

All patients were followed up after discharge with flexible bronchoscopies performed at 10 days, 1–3—6–12 months after surgery.

### Statistical analysis

Continuous variables are described as mean ± standard deviation (SD) and median (intra-quartile range, IQR). Categorical variables are reported as counts and percentages. Statistical analysis was performed on STATA 14.0 statistical software (StataCorp.2015. Stata Statistical Software: Release 14. College Station, TX: StataCorp LP).

## Results

**Figure 1:**
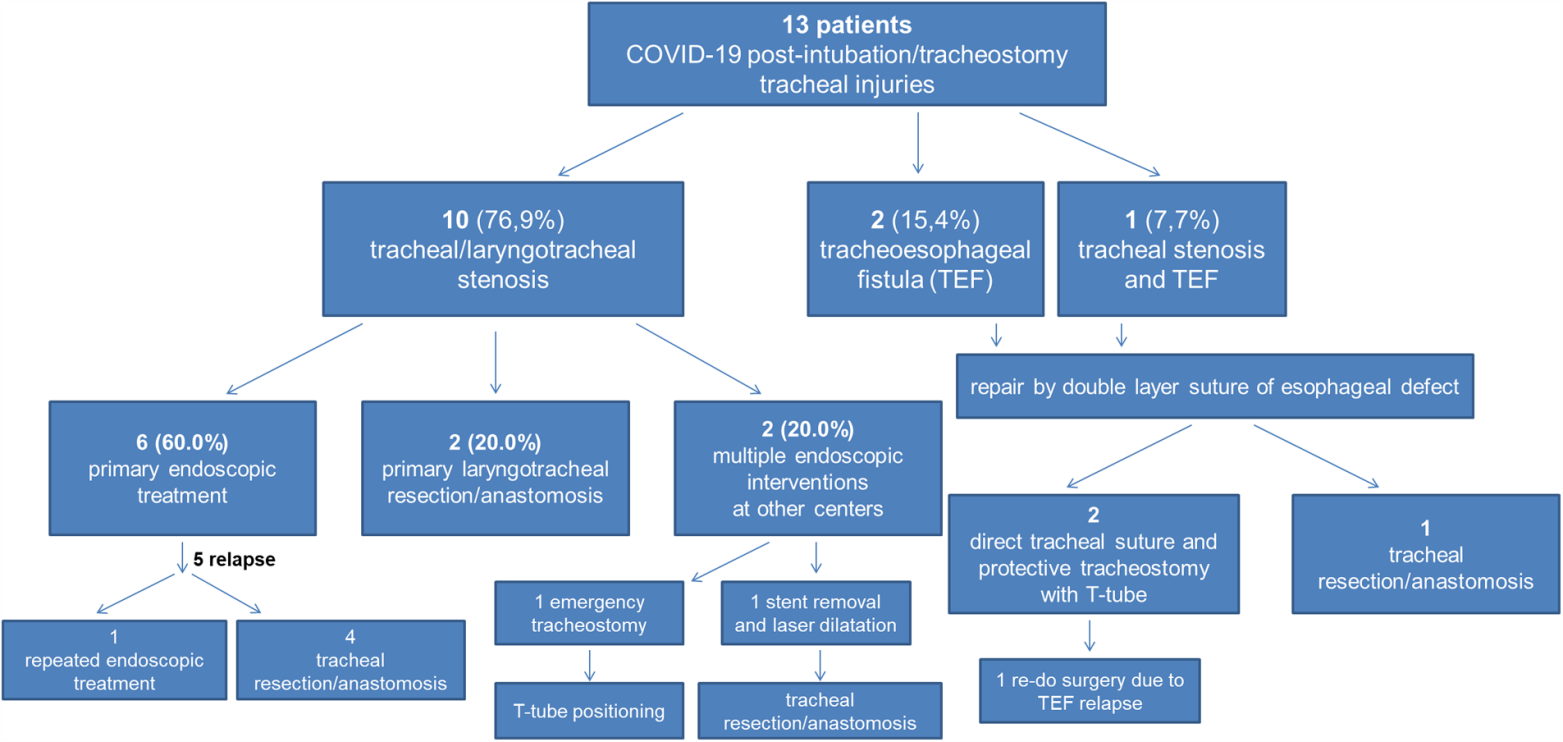
Treatment algorithm and outcomes in our patients with COVID-19 post-intubation/tracheostomy tracheal injuries.

A total of thirteen patients (8 males, 5 females) underwent endoscopic/surgical treatment for PI/T tracheal injuries after prolonged intubation and mechanical ventilation due to respiratory failure resulting from SARS-CoV-2 infection between March 2020 and February 2022. Particularly, 10 (76.9%) patients presented with tracheal/laryngotracheal stenosis, 2 (15.4%) with tracheoesophageal fistula (TEF) and 1 (7.7%) with concomitant TEF and stenosis. Age ranged from 37 to 76 years (mean 61.9 ± 12.2). The demographic and clinical characteristics of the patients are described in Table 1. Mean IMV duration (endotracheal tube and tracheostomy) was 47.0 ± 33.6 days. Eleven (84.6%) patients had undergone tracheostomy, with a mean duration of 49.2 ± 46.1 days. All patients diagnosed with TEF were already hospitalized and were sent to our Department to undergo emergency surgery; four patients (40.0%) with tracheal stenosis were admitted from the emergency department to our Unit to undergo urgent procedures and the last six patients (60.0%) were admitted to the outpatient clinic. Table 2 summarizes the bronchoscopic features of patients' tracheal stenosis and/or TEF. Figure 1 summarizes the treatment algorithm and outcomes.

**Table 1:** Demographic and clinical characteristics of the enrolled patients.

Variable	*n* (%)
**Gender**	
Female	5 (38.5)
Male	8 (61.5)
**Age** (years)	61.9 ± 12.2
**BMI** (kg/m^2^)	29.5 ± 4.7
**Comorbidities**	
COPD	5 (38.5)
Diabetes	1 (7.7)
Hypertension	5 (38.5)
Cardiopathy	2 (15.4)
Myasthenia Gravis	1 (7.7)
Cancer	1 (7.7)
Other	7 (53.8)
**Time from endotracheal intubation to tracheostomy** (days)	17.0 ± 5.4
**Tracheostomy** (*n*)	11 (84.6)
**Tracheostomy duration** (days)	49.2 ± 46.1; 33 (IQR 22;67.5)
**Duration of the IMV (endotracheal tube and tracheostomy)** (days)	47.0 ± 33.6; 43 (IQR 22;53)

**Table 2:** Bronchoscopic features of patients’ tracheal stenosis and/or TEF.

Patients with tracheal stenosis	
**Time from extubation to diagnosis** (days)	103.2 ± 73.1; 74.5 (IQR 56;137)
**Type of stenosis, *n* (%)**	
Web-like	4 (40.0)
Complex	6 (60.0)
**Localization *n* (%)**	
Subglottic	5 (50.0)
Upper 1/3 trachea	4 (40.0)
Middle trachea	1 (10.0)
**Residual lumen** (mm)	6.5 ± 1.65
**Number of endoscopic procedures**	20
**Type of procedure**	
Dilatation only	3 (30.0)
Surgery only	2 (20.0)
Dilatation + Surgery	5 (50.0)
Patients with TEF	
**Length of fistula** (cm)	3.0 ± 0.25
**Type of procedure**	
Primary oesophageal/tracheal closure	2 (66.7)
Tracheal resection/anastomosis + oesophageal closure	1 (33.3)

**Figure 2:**
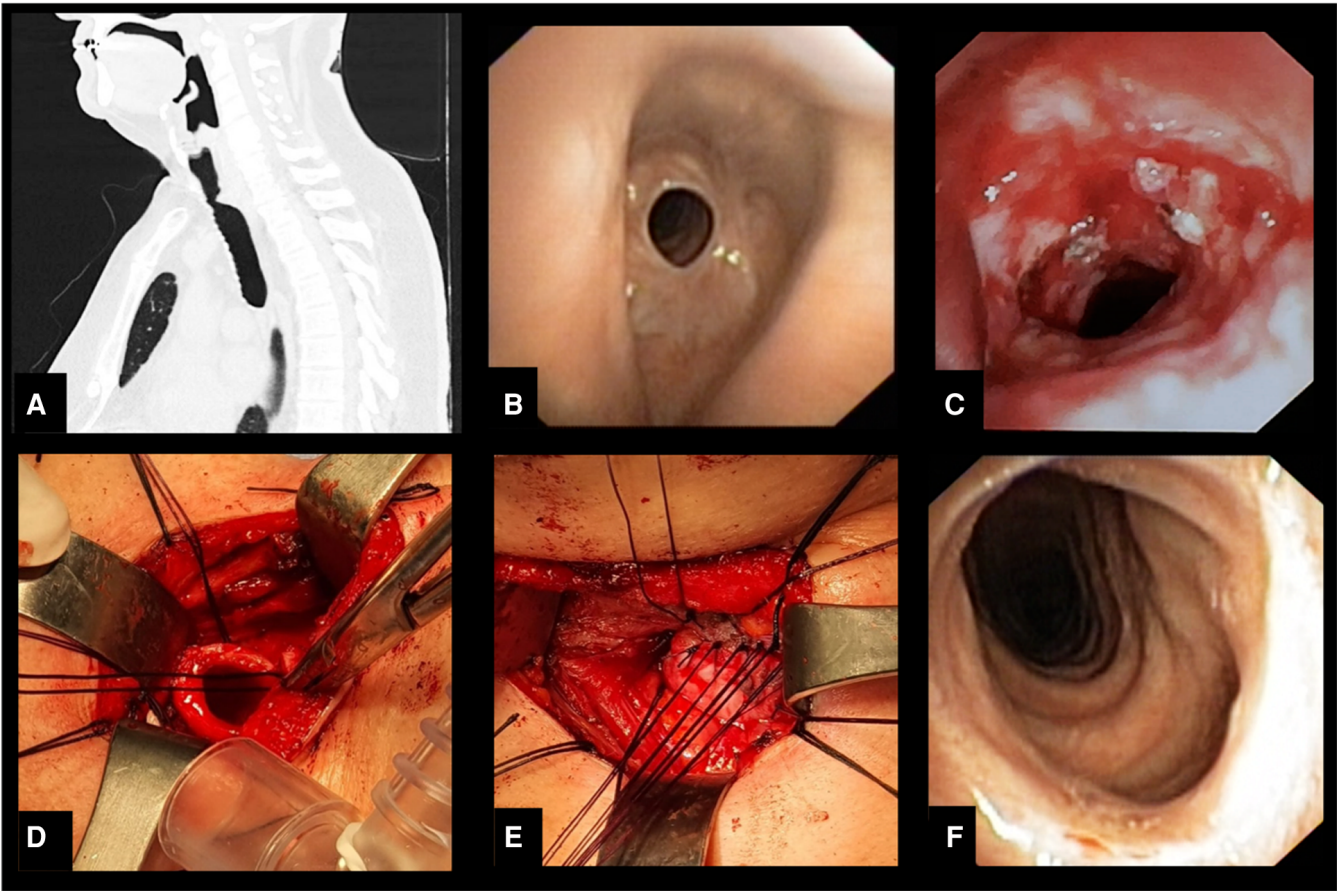
(**A**) Sagittal computed tomography image showing stenosis of the upper third of trachea. (**B**) Bronchoscopic appearance of the stenosis. (**C**) Endoscopic appearance of the recurrence of stenotic tracheal segment after endoscopic treatment with laser and dilation. (**D**) Intraoperative photo after resection of the stenotic tracheal segment. (**E**) Intraoperative appearance of the tracheal anastomosis. (**F**) Bronchoscopic appearance 3 months after surgery.

Among the ten patients with stenosis, two (20.0%) underwent primary laryngotracheal resection/anastomosis, two (20.0%) had undergone multiple endoscopic interventions before referral to our Centre: in one case, at arrival, emergent tracheostomy and T-tube positioning was performed; in the other one, a previously positioned endotracheal nitinol stent causing tracheal stenosis/granulation was removed and laser resection and tracheal dilatation was performed at first. Repeated rigid bronchoscopic procedures with tracheal dilatation were performed on this patient and, at least, post-treatment relapse required tracheal resection/anastomosis. Six (60.0%) patients were initially treated with rigid bronchoscopy procedures (laser and/or dilatation). Post-treatment relapse was experienced in 5 (50.0%) cases, requiring repeated rigid bronchoscopy procedures in 1 (10.0%) and surgery (tracheal resection/anastomosis) in 4 (40.0%) for definitive resolution of the stenosis (Figure 2).

Three patients with TEF underwent surgical repair by double layer suture of oesophageal defect associated with tracheal resection/anastomosis (1 case) or direct tracheal suture (2 cases) and protective tracheostomy with T-tube insertion.

Early postoperative complications developed in 2 cases (67%): one patient experienced a small (<5 mm) oesophageal suture leak in the immediate postoperative course, managed by using endoscopic through-the-scope clips (TTSC). The other patient, instead, experienced a TEF relapse successfully managed by redo-surgery. Particularly, the relapse was firstly managed through the endoscopic placement of a self-expanding covered nitinol stent (Ultraflex, Boston Scientific, MA, USA) without resolution of the fistula. Two more surgeries were then needed to perform a faringostomy and upper oesophageal exclusion by cervicotomy with protection tracheostomy with T-tube insertion. Definitive surgery was performed after three months, allowing complete oesophageal recanalization and tracheostomy closure.

During long-term follow-up, only one patient (33.3%) treated for TEF developed a minor complication, that is to say bacterial infection of the cervicotomy treated with local medications and systemic antibiotic therapy.

Early postoperative complications developed in 20.0% of patients who underwent TR/A; they experienced postoperative low-grade laryngeal oedema in the immediate postoperative course, successfully managed by low-dose systemic corticosteroids and delayed extubation. During long-term follow-up, only 1 case of the TR/A treated patients developed non-obstructive postoperative granulation tissue at the anastomosis, successfully regressed with medical therapy including inhaled beclometasone and low-dose systemic corticosteroids. In all the other cases, long-term follow-up highlighted no major complication.

Intraoperative and postoperative patients' features are summarized in Table 3.

**Table 3:** Intraoperative and postoperative patients’ features.

Endoscopic dilatation	*n* (%)
Mean duration of procedure (min)	36.3 ± 16.3
Tracheal resection/anastomosis	
Mean duration of operation (min)	252.9 ± 46.8
Postoperative ICU-stay (patients)	6 (85.7)
Postoperative ICU LOS (days)	1.6 ± 1.3
In-hospital LOS (days)	7.7 ± 0.5
Complications	
*Anastomotic*	
Granulation tissue	1 (14.3)
*Non-anastomotic*	
Laryngeal oedema	2 (28.6)
Closure of TEFs	
Time from diagnosis to surgery (days)	9.3 ± 6.7
Mean duration of operation (min)	261.7 ± 79.7
ICU-stay (patients)	2 (66.7)
ICU LOS (days)	5.3 ± 6.1
Postoperative LOS (days)	50.3 ± 34.6;
Complications	
Recurrent TEF	1 (33.3)
Dysphagia	2 (66.6)
Wound infection	1 (33.3)

The pathologic examination of the tracheal segments resected from the patients who underwent tracheal surgery revealed fibrosis, inflammation, degeneration and ischemic necrosis of the tracheal rings but, in comparison with other tracheal specimens resected from non-COVID-19 related patients, no particular differences were observed.

**Figure 3:**
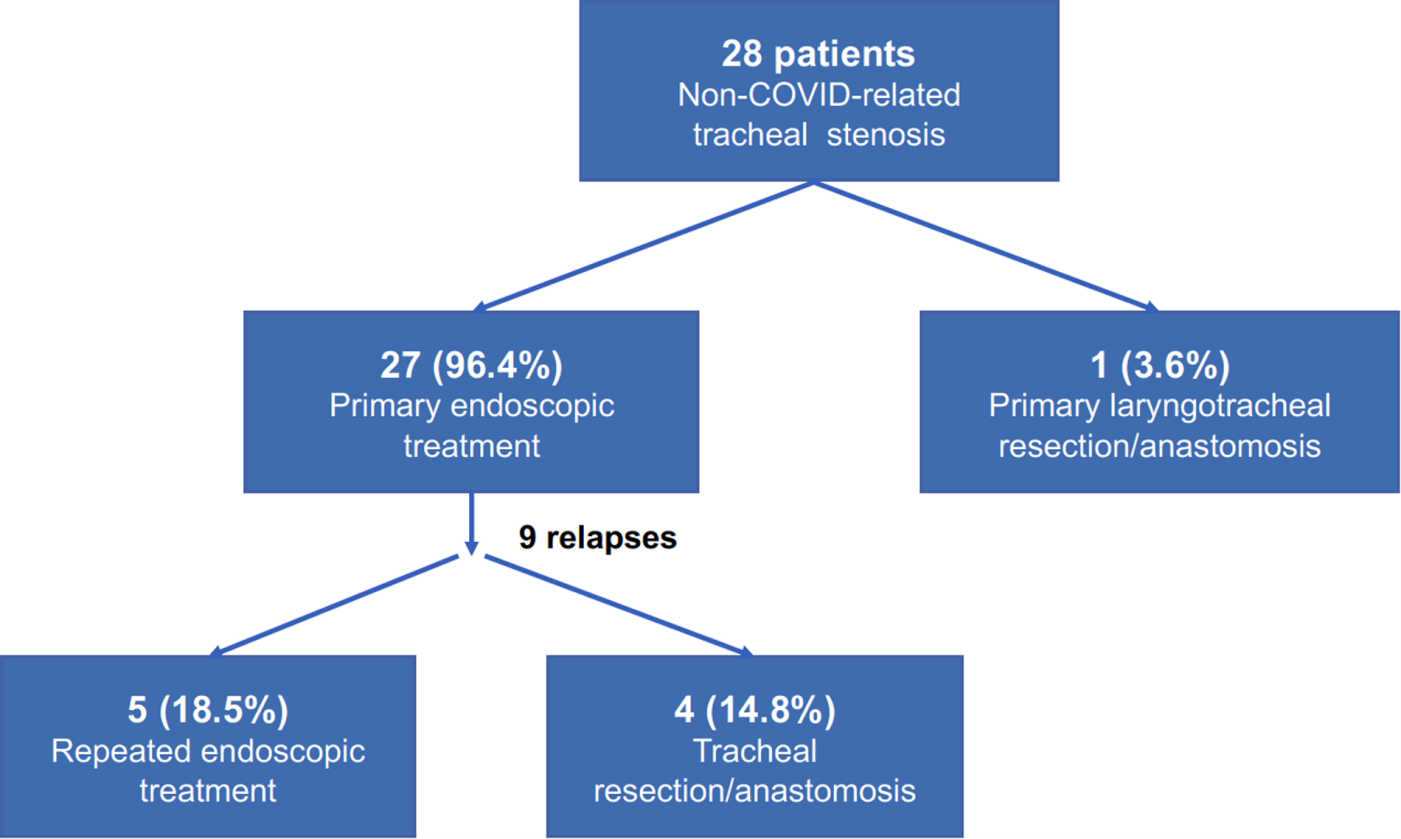
Treatment algorithm and outcomes in our patients with non-COVID-related tracheal injuries.

As an explorative analysis, the non-COVID-related PI/T treated at out Department in the same years were reviewed. A total of 28 patients had presented with tracheal stenosis, of them 7 (25%) patients presented with idiopathic stenosis and 21 (75%) with post-intubation/tracheostomy non-COVID-related stenosis. One (3.6%) patient underwent primary laryngotracheal resection/anastomosis, while the others 27 (96.4%) had undergone primary endoscopic treatment (laser and/or dilatation); among them, 3 (11.1%) patients had already undergone multiple endoscopic interventions before referral to our Centre. Post-treatment relapse was experienced in 9 (33.3%) cases, requiring repeated rigid bronchoscopy procedures in 5 (18.5%) and surgery (tracheal resection/anastomosis) in 4 (14.8%) for definitive resolution of the stenosis (Figure 3).

## Discussion

The recent COVID-19 pandemic has raised new medical and surgical challenges. The virus contagiousness, morbidity, and mortality have forced the clinicians to study new therapeutic options in the field of pneumology and infectious diseases, but surgery too has been forced to adapt to new scenarios and needs. As a consequence of prolonged IMV due to COVID-19 severe acute respiratory syndrome (SARS), in fact, a significant number of PI/T upper airways lesions has been detected, including both tracheal/laryngotracheal stenosis and tracheoesophageal fistulas. During the pandemic, around 10%–20% of infected patients have developed a severe disease requiring IMV for an average of 18 days (10–12) thus leading to an increase rate of upper airways lesions. Although the described incidence rates of tracheal stenosis following laryngotracheal intubation and tracheostomy range from 6% to 21% and 0.6% to 21% respectively in literature (13–17), recent studies have described the occurrence of PI/T lesions in almost half of the COVID-19 treated with prolonged IMV (18). Fiacchini et al. (19), in fact, compared 30 COVID-19 patients and 45 non-COVID-19 patients who both underwent prolonged IMV, and they found that 47% of COVID-19 patients developed PI/T upper airways complications compared to 2% of the control group. Many mechanisms have been regarded as responsible of the tracheal and oesophageal mucosal damage: the prothrombotic and antifibrinolytic state caused by SARS-CoV-2 virus producing microvascular injury and necrosis, high viral replication within the mucosa itself, chronic high dose systemic steroids use and the hypoxic damage and pronation manoeuvres used during the ICU stay which increase the pressure on the tracheal walls (1, 5, 6). Moreover, while generally tracheostomy should be performed 7 to 14 days after intubation to promote faster weaning and reduce IMV complications, during the COVID-19 period, tracheostomies were delayed until the patients were clear from the virus (3 to 4 weeks) because of the high risk of cross-infection of healthcare professionals, the need for continuation of prone position ventilation and the death of patients prior to the first 14 days. Also in our series, along with the worldwide records, in fact, mean duration time from intubation to tracheostomy was 17 days. Moreover, past case reports have shown times to onset from 28 days to 6 months (20). Shin et al. (21), in their study on 117 post-intubation tracheal lesions, reported a mean time from tracheal trauma to diagnosis of 1.8 months. Similarly, Beyoglu et al. (22) found a time to diagnosis of 42.0 days in the COVID-19 group with no statistically significant difference when comparing it with the non-COVID-19 group. In our case series, time from extubation to diagnosis was longer than the previous reports, around 100 days in mean.

Furthermore, a strict correlation between COVID-19, obesity and IMV has already been described (23) and, also in our study, most of patients were overweight with a mean BMI of 29.5. In our experience, in more than one case, surgical tracheal resection was delayed in favour of multiple endoscopic dilatations because of obesity, and patients were warned to lose weight before surgery. In COVID-19 population avoiding tracheal stenting has been suggested, because of its potential tissue damage on the airway wall causing an inflammatory response with granulation and causing delaying of surgical treatment (24). In our study, in fact, a patient had been admitted to our Centre with a previously positioned endotracheal nitinol stent for stenosis/granulation and, after removal, the inflammatory response of the tracheal wall had caused multiple relapses requiring multiple endoscopic dilatation and, at least, after maturation of the stenosis, tracheal resection and anastomosis was performed. Some recent reports have suggested that open surgical approaches should be avoided as primary choice in the COVID-19 population because of the high risk for comorbidities linked to the infection itself (25). In our experience, the distance from vocal cords did not influence the choice of treatment; half of our patients, in fact, had a subglottic stenosis and their treatment did not differ from the others. Type and grade of stenosis, instead, along with the patient characteristics, had guided the choice of treatment; in almost all cases an endoscopic dilatation attempt was made at first while upfront surgery was chosen for two patients with complex stenosis who had recovered from COVID-19 infection for at least 5 months and had developed a mature stenosis, in the absence of other serious comorbidities. Stratakos et al. (26) recently presented their case series on 23 COVID-19 patients diagnosed with post-intubation tracheal stenosis and TEF; in this study 65% of patients was initially treated with rigid bronchoscopic modalities and/or stent placement and 35% with tracheal resection-anastomosis, presenting rates similar to ours.

In literature, success rates of laser/mechanical dilatation of simple stenosis varies from 60% to 100% (27–29), with relapse rates after dilatation as high as 90%, especially in complex stenoses.

Cavaliere et al. (30) reported good results in 66% of benign tracheal stenosis cases treated with endoscopic manoeuvres, whereas Galluccio et al. (27) reported success rates of 96% and 69% for simple and complex stenoses, respectively. When looking at our results of the tracheal procedures in non-COVID patients, we experienced success rates of primary endoscopic procedures of approximately 67%. In our COVID-19-related case series, instead, recurrence developed in 6 out of 8 patients (75%) initially treated with mechanical tracheal dilatation and endoscopic dilatation was performed for a minimum of 1 and a maximum of 5 sessions due to symptomatic stenosis. It could be speculated that the pathologic alterations produced by the virus could impact on the capacity of the mucosa itself to heal properly because of the microvascular injury and the high viral replication within the mucosa itself. However, at the pathologic examinations, no particular differences were observed and more studies are needed to properly assess this issue.

In literature, TR/A complication rates range from 15% and 45% (22). In our study, only 1 patient presented small granulation tissue formation at the anastomotic site, which did not require any invasive treatment; laryngeal edema presenting in the immediate postoperative period was transient and regressed after 24–48 h.

TEF is a rare clinical entity, accounting for 0.5% of patients requiring prolonged IMV; recent series have proved an increase in its incidence during the COVID-19 pandemic, with rates increasing from 0.5 to 1.5% (31). Nonoperative mortality rate for TEFs is as high as 80% (32). In literature, many different approaches have been proposed for TEFs but, due to the rarity of this entity, no consensus exists, especially for the COVID-19 patients. Many authors have proposed conservative treatment for small TEFs, by temporarily bypassing the fistula with a tracheostomy tube, perform percutaneous jejunostomy and wait for healing, but all these approaches have showed high failure rates, always requiring definitive surgery (33–36). In our experience, all TEFs cases were managed urgently with surgery. Due both to the novelty and the rarity of TEF occurrence in COVID-19 patients, most of the published experience is anecdotal. Most of case reports (34–38) describe a conservative treatment, followed by delayed surgical repair of TEFs as the preferred option in COVID-19 patients. Gomez Zuleta et al. (31) described their series of 14 COVID-19 patients with TEF treated by either surgical (6 patients) or endoscopic (8 patients) repair; they found that 42.8% of the patients died due to infectious complications, with two patients dying before receiving surgical management. Palacios et al. (39), instead, described their series on 20 patients with TEF who were all managed by direct closure; only two patients (3.2%) developed dehiscence of the surgery site. Along with this last experience, we preferred timely surgical closure in all our TEF cases, since we feared that the virus could worsen ischemic damage of both the tracheal and oesophageal wall and enlarge the defect, preventing an effective surgical closure. Two out of three patients with TEF in our study experienced minor complications, all successfully managed with medical treatment. In one case, the patient experienced a primary failure of the surgical repair requiring a redo-surgery.

In conclusion, PI/T upper airways lesions during the COVID-19 pandemic have showed an increase in their incidence because of the longer need for IMV and the consequent delay of tracheostomies, the SARS-CoV-2 virus itself producing microvascular injury and necrosis, the high dose systemic steroids use and the hypoxic damage and pronation manoeuvres used during the ICU stay. Surgeons should be comfortable with all the therapeutic strategies, which do not differ from those for upper airways lesions in general. Firstly, the endoscopic evaluation is of paramount importance in the treatment plan, since the location, length, and severity of the stenosis or damages should guide the proper surgical or endoscopic management. Secondly, surgery should always be considered, when feasible, both for tracheal stenosis and for tracheoesophageal fistulas. TEFs in particular should always be treated as soon as possible, since the virus itself could weak and damage tracheal and oesophageal wall, preventing future surgery.

## Data Availability

The raw data supporting the conclusions of this article will be made available by the authors, without undue reservation.

## References

[1] Huang C Wang Y Li X Ren L Zhao J Hu Y Clinical features of patients infected with 2019 novel coronavirus in Wuhan, China. Lancet. (2020) 395:497–506. 10.1016/S0140-6736(20)30183-5 31986264 PMC7159299

[2] Sue RD Susanto I. Long-term complications of artificial airways. Clin Chest Med. (2003) 24:457–71. 10.1016/S0272-5231(03)00048-0 14535220

[3] Wood DE Mathisen DJ. Late complications of tracheotomy. Clin Chest Med. (1991) 12:597–609. 10.1016/S0272-5231(21)00806-6 1934960

[4] Wain JC Jr. Postintubation tracheal stenosis. Semin Thorac Cardiovasc Surg. (2009) 21:284-9. 10.1053/j.semtcvs.2009.08.001 19942129

[5] Minonishi T Kinoshita H Hirayama M Kawahito S Azma T Hatakeyama N The supine-to-prone position change induces modification of endotracheal tube cuff pressure accompanied by tube displacement. J Clin Anesth. (2013) 25:28–31. 10.1016/j.jclinane.2012.05.007 23391343

[6] Magro C Mulvey JJ Berlin D Nuovo G Salvatore S Harp J Complement associated microvascular injury and thrombosis in the pathogenesis of severe COVID-19 infection: a report of five cases. Transl Res. (2020) 220:1–13. 10.1016/j.trsl.2020.04.007 32299776 PMC7158248

[7] Bradley BT Maioli H Johnston R Chaudhry I Fink SL Xu h Histopathology and ultrastructural findings of fatal COVID-19 infections in Washington state: a case series. Lancet. (2020) 396:320–32. 10.1016/S0140-6736(20)31305-2 32682491 PMC7365650

[8] Scholfield DW Warner E Ahmed J Ghufoor K. Subglottic and tracheal stenosis associated with coronavirus disease 2019. J Laryngol Otol. (2021) 135:656–8. 10.1017/S0022215121001134 33973511

[9] Marulli G Rizzardi G Bortolotti L Loy M Breda C Hamad AM Single-staged laryngotracheal resection and reconstruction for benign strictures in adults. Interact Cardiovasc Thorac Surg. (2008) 7:227–30. 10.1510/icvts.2007.168054 18216046

[10] Marulli G Loizzi M Cardillo G Battistella L De Palma A Ialongo P Early and late outcome after surgical treatment of acquired non-malignant tracheo-oesophageal fistulae. Eur J Cardio-Thor Surg. (2013) 43:e155–61. 10.1093/ejcts/ezt069 23444410

[11] Senturk M El Tahan MR Szegedi LL Marczin N Karzai W Shelley B Thoracic anesthesia of patients with suspected or confirmed 2019 novel coronavirus infection: preliminary recommendations for airway management by the European association of cardiothoracic anaesthesiology thoracic subspecialty committee. J Cardiothorac Vasc Anesth. (2020) 34:2315–27. 10.1053/j.jvca.2020.03.059 32414544 PMC7151284

[12] Grasselli G Zangrillo A Zanella A Antonelli M Cabrini L Castelli A COVID-19 Lombardy ICU network. Baseline characteristics and outcomes of 1591 patients infected with SARS-CoV-2 admitted to ICUs of the lombardy region, Italy. JAMA. (2020) 323:1574–81. 10.1001/jama.2020.5394 32250385 PMC7136855

[13] Elsayed HH Hassaballa AS Ahmed TA Gumaa M Sharkawy HY Moharram AA. Variation in outcome of invasive mechanical ventilation between different countries for patients with severe COVID-19: a systematic review and meta-analysis. PLoS One. (2021) 16:e0252760. 10.1371/journal.pone.0252760 34086779 PMC8177443

[14] Sarper A Ayten A Eser I Ozbudak O Demircan A. Tracheal stenosis aftertracheostomy or intubation: review with special regard to cause and management. Tex Heart Inst J. (2005) 32:154–8. PMID: . 16107105 PMC1163461

[15] Grillo HC Donahue DM Mathisen DJ Wain JC Wright CD. Postintubation tracheal stenosis. Treatment and results. J Thorac Cardiovasc Surg. (1995) 109:486–92. 10.1016/S0022-5223(95)70279-2 7877309

[16] Diaz-Jimenez JP Lisbona RML. Benign tracheal and bronchial stenosis. In: Díaz-Jimenez JP Lisbona RML, editors. Interventions in pulmonary medicine. New York: Springer (2013). p. 165–184.

[17] Papla B Dyduch G Frasik W Olechnowicz H. Post-intubation tracheal stenosis–morphological-clinical investigations. Pol J Pathol. (2003) 54:261–6. PMID: . 14998295

[18] Esteller-More E Ibanez J Matino E Adema JM Nolla M Quer IM. Prognostic factors in laryngotracheal injury following intubation and/or tracheotomy in ICU patients. Eur Arch Otorhinolaryngol. (2005) 262:880–3. 10.1007/s00405-005-0929-y 16258758

[19] Fiacchini G Tricò D Ribechini A Forfori F Brogi E Lucchi M Evaluation of the incidence and potential mechanisms of tracheal complications in patients with COVID-19. JAMA Otolaryngol Head Neck Surg. (2021) 147:70–6. 10.1001/jamaoto.2020.4148 33211087 PMC7677875

[20] Liu J Zhang CP Li Y Dong S. Post-intubation tracheal stenosis after management of complicated aortic dissection: a case series. J Cardiothorac Surg. (2015) 10:148. 10.1186/s13019-015-0357-z 26537875 PMC4632458

[21] Shin B Kim K Jeong BH Eom JS Song WJ Kim H. Clinical implications of differentiating between types of post-tracheostomy tracheal stenosis. J Thorac Dis. (2017) 9:4413–23. 10.21037/jtd.2017.10.99 29268510 PMC5721046

[22] Beyoglu MA Sahin MF Turkkan S Yazicioglu A Yekeler E. Complex post-intubation tracheal stenosis in COVID-19 patients. Indian J Surg. (2022) 84:805–13. 10.1007/s12262-022-03498-x 35818393 PMC9261125

[23] Simonnet A Chetboun M Poissy J Raverdy V Noulette J Duhamel A High prevalence of obesity in severe acute respiratory syndrome coronavirus-2 (SARS-CoV-2) requiring invasive mechanical ventilation. Obesity (Silver Spring). (2020) 28:1195–9. 10.1002/oby.22831 32271993 PMC7262326

[24] Gaissert HA Grillo HC Wright CD Donahue DM Wain JC Mathisen DJ. Complication of benign tracheobronchial strictures by self-expanding metal stents. J Thorac Cardiovasc Surg United States. (2003) 126:744–7. 10.1016/S0022-5223(03)00361-1 14502148

[25] Mattioli F Marchioni A Andreani A Cappiello G Fermi M Presutti L. Post-intubation tracheal stenosis in COVID-19 patients. Eur Arch Otorhinolaryngol. (2021) 278:847–8. 10.1007/s00405-020-06394-w 33011955 PMC7532739

[26] Stratakos G Anagnostopoulos N Alsaggaf R Koukaki E Bakiri K Emmanouil P COVID-19 Patients presenting with post-intubation upper airway complications: a parallel epidemic? J Clin Med. (2022) 11:1719. 10.3390/jcm11061719 35330044 PMC8948992

[27] Galluccio G Lucantoni G Battistoni P Paone G Batzella S Lucifora V Interventional endoscopy in the management of benign tracheal stenoses: definitive treatment at long-term follow-up. Eur J Cardiothorac Surg. (2009) 35:429–33. 10.1016/j.ejcts.2008.10.041 19084420

[28] Dalar L Karasulu L Abul Y Ozdemir C Sokucu SN Tarhan M Bronchoscopic treatment in the management of benign tracheal stenosis: choices for simple and complex tracheal stenosis. Ann Thorac Surg. (2016) 101:1310–7. 10.1016/j.athoracsur.2015.10.005 26704411

[29] Dutau H Breen DP. Endobronchial laser treatment: an essential tool in therapeutic bronchoscopy. Eur Respir Monogr. (2010) 48:149–60.

[30] Cavaliere S Bezzi M Toninelli C Foccoli P. Management of post-intubation tracheal stenoses using the endoscopic approach. Monaldi Arch Chest Dis. (2007) 67:73–80. 10.4081/monaldi.2007.492 17695689

[31] Gomez Zuleta MA Gallego Ospina DM Ruiz OF. Tracheoesophageal fistulas in coronavirus disease 2019 pandemic: a case report. World J Gastrointest Endosc. (2022) 14:628–35. 10.4253/wjge.v14.i10.628 36303807 PMC9593510

[32] Gaissert HA Lofgren RH Grillo HC. Upper airway compromise after inhalation injury. Complex strictures of the larynx and trachea and their management. Ann Surg. (1993) 218:672. 10.1097/00000658-199321850-00014 8239783 PMC1243040

[33] Roomi S Talib U Farooq S Chohan A Siddique M Saeed R Tracheoesophageal fistula: a rare complication of prolonged intubation in COVID-19. Chest. (2020) 158:A2596–7. 10.1016/j.chest.2020.09.191

[34] Pereira C Silva R Campello GC Moura F. Tracheoesophageal fistula in a COVID-19 patient. Saudi J Anaesth. (2021) 15:447–9. 10.4103/sja.sja_294_21 34658736 PMC8477781

[35] Negaresh M Hoseininia S Samadi Takaldani AH Feyzi-Khankandi I Mohammadzadeh Germi B Ghobadi H. Implanting jejunostomy tube as conservative management of tracheoesophageal fistula in a COVID-19 patient. Clin Case Reports. (2021) 9:e05151. 10.1002/ccr3.5151 PMC8643485 34917369

[36] Cuaño PMGM Pilapil JCA Larrazabal RJB Villalobos RE. Acquired tracheoesophageal fistula in a pregnant patient with COVID-19 pneumonia on prolonged invasive ventilation. BMJ Case Rep. (2021) 14:e244016. 10.1136/bcr-2021-244016 PMC8381298 34417243

[37] Rosati R De Nardi P Dell'Acqua A Calvi MR Elmore U Scarparo E Tracheoesophageal Fistula in a COVID-19 ventilated patient: a challenging therapeutic decision. Case Rep Surg. (2021):6645518. 10.1155/2021/6645518 33833892 PMC8014238

[38] Granata A Martucci G Rizzo GEM Arcadipane A Traina M. Combined endoscopical treatments for tracheo-esophageal fistula developed during V-V ECMO for severe COVID-19: a case series. Artif Organs. (2022) 46:506–8. 10.1111/aor.14101 34755351 PMC8653176

[39] Palacios JM Bellido DA Valdivia FB Ampuero PA Figueroa CF Medina C Tracheal stenosis as a complication of prolonged intubation in coronavirus disease 2019 (COVID-19) patients: a Peruvian cohort. J Thorac Dis. (2022) 14:995–1008. 10.21037/jtd-21-1721 35572866 PMC9096309

